# Burden and Predictors of Statin Use for Primary and Secondary Prevention of Cardiovascular Disease in Bangladesh: Evidence from a Nationally Representative Survey

**DOI:** 10.5334/gh.1412

**Published:** 2025-03-12

**Authors:** Shehab Uddin Al Abid, Md Mostafa Monower, Ahmad K. Abrar, Jannat A. Riva, Mahfuzur Rahman Bhuiyan, Mohammad Abdullah Al-Mamun, Sohel Reza Choudhury

**Affiliations:** 1 Department of Epidemiology & Research, National Heart Foundation Hospital and Research Institute, Dhaka, Bangladesh; 2 Ad-din Women’s Medical College Hospital, Outer Circular Rd, Dhaka 1217, Bangladesh

**Keywords:** statins, cardiovascular disease, Bangladesh, Primary prevention, ACC/AHA, WHO

## Abstract

**Background:**

Large-scale randomized trials have established the efficacy and safety of statin therapy in preventing cardiovascular diseases (CVDs) among individuals at increased risk (i.e., primary prevention) or those with pre-existing cardiovascular disease (i.e., secondary prevention). Consequently, recent international guidelines, including those from the WHO and ACC/AHA, have expanded the eligibility criteria for statin therapy.

**Objective:**

To assess the current burden of statin-eligible populations in Bangladesh, evaluate the current state of statin use, and identify factors associated with non-use of statins.

**Methods:**

We analysed data from 3,140 adults aged 40 to 69 years from the nationally representative WHO-STEPS Bangladesh 2018 survey. Statin therapy eligibility for primary prevention was assessed using the WHO-2019 and the ACC/AHA-2018 guidelines separately. Individuals with a previous history of CVD were eligible for secondary prevention under both guidelines. Modified Poisson regression models identified factors associated with statin use. All analyses were conducted using appropriate survey weights.

**Findings:**

Among the participants, 443 (14.1%) reported a previous history of CVD. Of those without CVD, 11.2% (95% CI: 9.7–12.9) and 32.3% (95% CI: 30.0–34.6) were eligible for statin use for primary prevention according to the WHO-2019 and the ACC/AHA-2018 guidelines, respectively. Among adults eligible according to WHO-2019 guideline, 6.9% (95% CI: 4.1–11.5) were using statins, while among those eligible according to ACC/AHA-2018 guideline, 3.3% (95% CI: 2.1–5.1) were using statins. For secondary prevention, 23.5% (95% CI: 16.9–31.6) of adults with prior CVD were using statins. Non-use was higher among younger adults, those without regular health visits or cholesterol measurements, and those from the Mymensingh or Rajshahi divisions.

**Interpretation:**

In Bangladesh, approximately one in twenty eligible individuals uses statins for primary prevention of CVD, and one in five individuals for secondary prevention. Appropriate population health interventions are needed to scale up statin use to mitigate the burden of CVD.

## Introduction

Over recent decades, cardiovascular diseases (CVDs) have remained the leading cause of death worldwide, accounting for an estimated 17.9 million deaths in 2019, which represents 32% of all global deaths (1). While age-standardized CVD mortality rates have consistently declined in many high-income countries (HICs), they have continued to rise in low- and middle-income countries (LMICs), including Bangladesh (2).

Hydroxymethylglutaryl coenzyme A reductase inhibitors, commonly known as statins, are among the most effective and safe pharmacological interventions for the prevention and treatment of CVDs (3,4). Evidence from large-scale randomized controlled trials has shown that each 1 mmol/L reduction in low-density lipoprotein cholesterol (LDL-C) achieved through statin therapy reduces the risk of major cardiovascular events—such as myocardial infarctions, cardiovascular deaths, and strokes—by approximately 25%, regardless of age, sex, or other comorbidities (3,4). Consequently, recent global guidelines, such as those from the World Health Organization (WHO) and the American College of Cardiology/American Heart Association (ACC/AHA), have expanded the eligibility criteria for statin therapy to prevent major cardiovascular events among individuals at increased risk (5,6).

However, recent reports suggest that, despite the proven efficacy of statins, their adoption varies widely between HICs and LMICs (7). In many HICs, more than 50% of eligible individuals are currently on statin therapy (7,8); whereas in many LMICs, only about 10% of eligible individuals use statin therapy for CVD prevention (9,10).

Bangladesh, an LMIC in South Asia with a population of over 170 million (11), currently has one of the highest rates of CVD mortality and morbidity in the world (12). However, the extent of statin use among eligible individuals remains unknown. It is unclear how many individuals meeting the criteria for statin therapy, based on international guidelines, are actually receiving it, and what factors contribute to statin use. Moreover, different guidelines use their own set of CVD risk assessment tools and thresholds for determining statin eligibility (5,6), which can significantly alter the total number of individuals eligible for statin therapy and have substantial implications for the country’s health budget. Addressing this knowledge gap is essential for informing public health interventions aimed at reducing the growing burden of CVD in Bangladesh.

The present study aims to assess the current burden of statin-eligible populations in Bangladesh according to two different international guidelines, evaluate the prevalence of statin use among these eligible populations, and identify the factors associated with non-use using the data of Bangladesh WHO-STEPS survey-2018 (13). By providing a nationally representative assessment, this study will contribute valuable evidence to guide policy and practice towards optimizing statin use for CVD prevention in Bangladesh.

## Methods

### Study design and participants

In this cross-sectional study, we used data from the 2018 WHO STEPwise approach to NCD risk factor surveillance (STEPS) survey of Bangladesh (13). The STEPS-Bangladesh 2018 survey was a nationally representative, population-based survey conducted from February to May 2018, encompassing adults aged 18 to 69 years. This is the most recent survey containing comprehensive data necessary to determine statin eligibility and usage.

A detailed description of the sampling technique for the STEPS-Bangladesh 2018 survey has been published elsewhere (13). Briefly, a two-stage, geographically stratified, probability-based sampling method was employed using the primary sampling units (PSUs) developed by the Bangladesh Bureau of Statistics (BBS) for the national census. In the first stage, 496 PSUs were selected from urban and rural areas across all eight administrative divisions of Bangladesh. One PSU was excluded due to inaccessibility, resulting in a total of 495 PSUs for the final survey. In the second stage, a fixed number of households (n = 20) was selected from each PSU using systematic random sampling. One eligible adult aged 18 to 69 years was randomly chosen for participation from each selected household (13,14). The sample size for the STEPS-Bangladesh 2018 survey was calculated using the WHO-recommended sample size calculator to ensure the generalisability and reliability of the findings to the entire target population of Bangladesh (13).

For the present study, we included all men and women aged 40 to 69 years who provided biological samples (n = 3, 246) in the STEPS-Bangladesh 2018 survey. This age range was selected because both the WHO and AHA/ACC guidelines and cardiovascular disease (CVD) risk assessment tools are applicable only to adults aged 40 years and older (5,6). Additionally, we excluded participants with triglyceride levels above 400 mg/dL (15), or those with missing blood pressure measurements (n = 106), as these data are necessary for calculating 10-year CVD risk scores **(Supplemental Figure 1)**.

The ethical approval for the Bangladesh WHO-STEPS 2018 survey was obtained from the Bangladesh Medical and Research Council. Informed written consent was obtained from each respondent before the interview and specimen collection (13).

### Data collection and procedures

Participants provided detailed socio-demographic, lifestyle, and health-related information through a structured interview. Blood pressure was measured using the “BP–BOSO–Medicus Control” digital monitor, validated by the German Hypertension League (14). Three consecutive blood pressure readings were taken, and the mean of the last two readings was used for the analysis.

Fasting blood samples were collected to measure blood glucose and lipid levels. Samples were obtained under strict aseptic conditions and transported to the laboratory within 24 hours. Rigorous quality control measures, including instrument calibration and cross-validation with a standard national laboratory, were implemented to ensure the accuracy of the biochemical results (13). Total cholesterol, triglycerides, and high-density lipoprotein cholesterol (HDL-C) were measured directly in the laboratory, while low-density lipoprotein cholesterol (LDL-C) levels were calculated using the Friedewald equation (not valid for triglyceride levels >400 mg/dL) (15).

### Statin eligibility and use

Eligibility for statin therapy for primary prevention (among individuals without a previous history of CVD) was determined separately according to the WHO and ACC/AHA guidelines. According to the WHO guideline, individuals with a history of diagnosed diabetes or a 10-year CVD risk greater than 20%, as assessed by the 2019 WHO laboratory-based risk equations (16), were eligible for statin therapy for primary prevention (5). The WHO 2019 risk model for cardiovascular disease is based on an individual’s age, sex, smoking status, systolic blood pressure, history of diabetes, and total cholesterol level (16).

Participants eligible for primary prevention according to the ACC/AHA guideline were: (1) individuals with LDL-C levels ≥190 mg/dL, (2) individuals with a history of diagnosed diabetes, or (3) individuals with LDL-C levels between 70 and 189 mg/dL and a predicted 10-year risk of any cardiovascular disease ≥7.5%, based on the new pooled cohort equations (6). South Asian ancestry has been identified as a ‘risk-enhancing’ factor for CVD by the ACC/AHA, and therefore, recommends a lower threshold of predicted CVD risk (7.5%, as opposed to the WHO’s 20%) to initiate statin therapy for primary prevention (6). Individuals with a prior history of CVD, irrespective of LDL-C levels, were considered eligible for secondary prevention according to both guidelines (5,6) **(Supplemental Table 1)**.

Outcomes of this analysis were the proportion of statin users for the primary and secondary prevention of CVD among those eligible according to the WHO and ACC/AHA guidelines. Participants who answered ‘yes’ to the following question in the STEPS survey were categorized as statin users: ‘Are you currently taking statins regularly to prevent or treat heart disease?’ (9,13).

### Statistical analysis

We first determined the proportion of participants eligible for statin therapy for the primary and secondary prevention of CVD based on the WHO 2019 and ACC/AHA 2018 guidelines, stratified by different socio-demographic characteristics. We then calculated the proportion of individuals using statins for primary and secondary prevention among those eligible for statin therapy according to these guidelines.

To calculate the 10-year CVD risk scores using the 2019 WHO laboratory-based equations, we used the “*whocvdrisk”* package in Stata, developed by the authors of the WHO-CVD risk prediction models and tailored for the Bangladeshi population (16). The ACC/AHA 2018 10-year CVD risk scores were determined using the pooled risk equation with the *“pooledcohort”* package in R software (17).

Kappa statistics were used to determine the level of agreement between the WHO and ACC/AHA guidelines for identifying participants eligible for statin therapy (18). As suggested by Landis and Koch (19), Kappa values were categorised as follows: <0.40 indicating poor to fair agreement, 0.41 to 0.60 as moderate agreement, 0.61 to 0.80 as substantial agreement, and 0.81 to 1.0 as almost perfect agreement.

To identify predictors of statin use among individuals eligible for statin therapy, we used modified Poisson regression models and reported risk ratios (RR) with 95% confidence intervals (CI) (20). The regression analysis was conducted only among individuals eligible for secondary prevention due to the low number of individuals using statins for primary prevention. We performed both partially and fully adjusted modified Poisson regression models to examine changes in effect sizes due to covariate adjustments. The partially adjusted model accounted for age (40–54 years, 55–69 years) and sex (male, female). The fully adjusted model additionally controlled for education level (no schooling, primary, secondary, college/higher), residence (rural, urban), division (Dhaka, Barishal, Chattogram, Khulna, Mymensingh, Rajshahi, Rangpur, Sylhet), regular health visits (yes, no), and history of cholesterol measurement (yes, no). All models adhered to Poisson regression assumptions.

In all analyses, we accounted for the complex survey design. All P-values were two-sided, with statistical significance set at <0.05. Analyses were performed using Stata version 17 (StataCorp, TX, United States) and R version 4.3.0 (R Core Team, Vienna, Austria).

## Results

A total of 3,140 participants were included in the analysis. The mean age was 51.1 years (standard deviation [SD]: 7.8), and 49.3% were women. Overall, 443 participants (14.1%) reported a history of cardiovascular disease (CVD). The predicted 10-year cardiovascular risk was generally higher among male participants than female participants, based on both the WHO and ACC/AHA risk equations. Female participants were more likely to report regular visits to health facilities (60.4%) compared to male participants (38.3%). Overall, 789 (23.8%) and 1,329 participants (41.9%) were eligible for statin use according to the WHO-2019 and ACC/AHA-2018 guidelines, respectively (Table 1).

**Table 1:** Study characteristics, overall and by sex.


CHARACTERISTIC	FEMALE N = 1,531	MALE N = 1,609	TOTAL N = 3140	P-VALUE^‡^

**Age, years**	49.9 (7.4)	52.3 (8.1)	51.1 (7.8)	<0.001

**Age**				<0.001

40–54 years	1,137 (71.4%)	996 (57.7%)	2,133 (64.4%)	

55–69 years	394 (28.6%)	613 (42.3%)	1,007 (35.6%)	

**Education**				<0.001

No schooling	820 (62.4%)	678 (48.4%)	1,498 (55.3%)	

Primary	542 (31.8%)	560 (33.9%)	1,102 (32.9%)	

Secondary	109 (4.4%)	249 (13.0%)	358 (8.8%)	

College/Higher	45 (1.4%)	122 (4.7%)	167 (3.1%)	

**Residence**				0.3

Rural	830 (81.5%)	857 (80.1%)	1,687 (80.8%)	

Urban	701 (18.5%)	752 (19.9%)	1,453 (19.2%)	

**Division**				0.7

Dhaka	205 (6.6%)	223 (6.6%)	428 (6.6%)	

Barishal	156 (19.4%)	195 (18.8%)	351 (19.1%)	

Chattogram	150 (19.2%)	167 (21.9%)	317 (20.6%)	

Khulna	211 (12.0%)	216 (12.2%)	427 (12.1%)	

Mymensingh	226 (11.4%)	220 (10.2%)	446 (10.8%)	

Rajshahi	218 (15.1%)	212 (13.8%)	430 (14.5%)	

Rangpur	203 (10.7%)	201 (11.1%)	404 (10.9%)	

Sylhet	162 (5.6%)	175 (5.2%)	337 (5.4%)	

**Regular health visit**				<0.001

Yes	945 (60.4%)	652 (38.3%)	1,597 (49.2%)	

No	586 (39.6%)	957 (61.7%)	1,543 (50.8%)	

**Ever measured cholesterol**				0.10

Yes	110 (5.2%)	147 (7.0%)	257 (6.1%)	

No	1,421 (94.8%)	1,462 (93.0%)	2,883 (93.9%)	

**Previous history of CVD**				0.2

No	1,305 (84.6%)	1,392 (87.1%)	2,697 (85.9%)	

Yes	226 (15.4%)	217 (12.9%)	443 (14.1%)	

**10-year CVD risk (WHO-2019)**	3.8 (3.1)	6.3 (4.4)	5.1 (4.0)	<0.001

**10-year CVD risk (ACC/AHA-2018)**	2.9 (3.6)	9.4 (8.2)	6.2 (7.1)	<0.001

**Eligible for statin use (WHO-2019)**				0.2

No	1,148 (74.7%)	1,203 (77.7%)	2,351 (76.2%)	

Yes	383 (25.3%)	406 (22.3%)	789 (23.8%)	

**Eligible for statin use (ACC/AHA-2018)**				<0.001

No	1,077 (70.6%)	734 (46.0%)	1,811 (58.1%)	

Yes	454 (29.4%)	875 (54.0%)	1,329 (41.9%)	


*Notes:* Data are presented as mean (SD) for continuous measures and n(unweighted) (%) for categorical measures. ^‡^Design-based t-test; Pearson’s X^2: Rao & Scott adjustment. ACC, American College of Cardiology; AHA, American Heart Association; N, number of participants; SD, standard deviation; WHO, World Health Organization.

**Figure 1:**
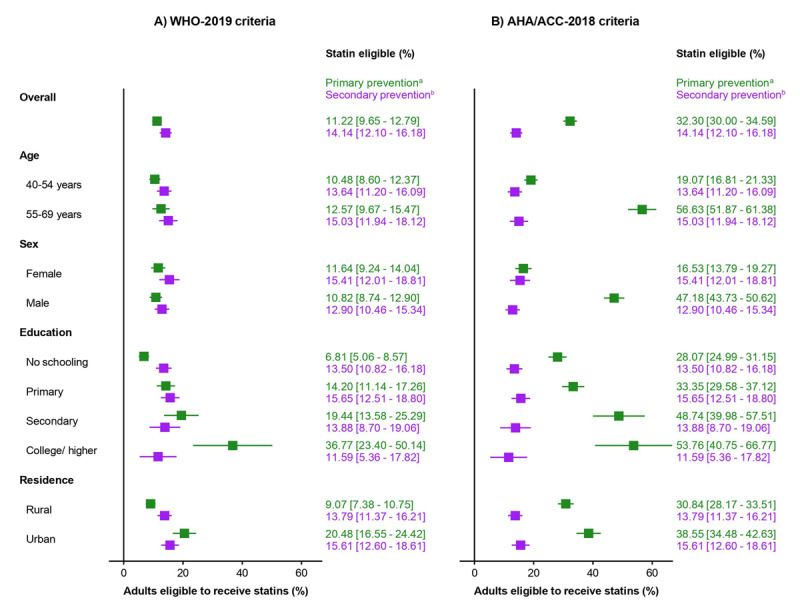
Proportion of Bangladeshi adults eligible for statin therapy according to the WHO-2019 and ACC/AHA-2018 guidelines, by primary and secondary prevention categories. ^a^Analysis of the primary prevention category was performed among individuals without cardiovascular disease. ^b^Only individuals with a history of cardiovascular disease were eligible for secondary prevention in both the guidelines. Abbreviations: ACC, American College of Cardiology; AHA, American Heart Association; WHO, World Health Organization.

Among participants without a history of CVD, 11.2% (95% confidence interval [CI]: 9.7–12.9) were eligible for statin therapy for primary prevention according to the WHO-2019 guideline, while 32.3% (95% CI: 30.0–34.6) were eligible under the ACC/AHA-2018 guideline. For secondary prevention, 14.1% (95% CI: 12.1–16.2) of all participants were eligible for statin therapy according to both guidelines (Figure 1).

According to the WHO-2019 guideline, the proportion of adults eligible for statin therapy was relatively consistent across age groups and between men and women. However, there were substantial differences by educational level and place of residence. Only 6.8% (95% CI: 5.1–8.6) of adults with no schooling were eligible for statin therapy for primary prevention, compared to 36.8% (95% CI: 23.4–50.1) of adults with a college degree or higher. Similarly, 20.5% (95% CI: 16.6–24.4) of the urban population were eligible, compared to 9.1% (95% CI: 7.4–10.8) of the rural population. In contrast, the ACC/AHA-2018 guideline showed marked differences in eligibility. For example, 56.6% (95% CI: 51.9–61.4) of adults aged 55–69 years were eligible, compared to 19.1% (95% CI: 16.8–21.3) of those aged 40–54 years. Additionally, 47.2% (95% CI: 43.7–50.6) of male participants were eligible, compared to 16.5% (95% CI: 13.8–19.3) of female participants (Figure 1).

All 346 participants eligible for primary prevention according to the WHO-2019 guideline were also eligible under the ACC/AHA-2018 guideline. An additional 540 participants were eligible for primary prevention only under the ACC/AHA-2018 guideline. There was moderate agreement between the two guidelines in identifying participants eligible for statin therapy for primary prevention (Kappa coefficient: 0.46). For secondary prevention, the agreement was perfect (Kappa coefficient: 1.00), as the eligibility criteria were identical in both guidelines (Table 2). Age- and sex-stratified analyses showed that younger females (aged 40–54 years) had almost perfect agreement (Kappa coefficient: 0.90), while older females and younger males had moderate agreement (Kappa coefficients: 0.55 and 0.45, respectively). However, older males (aged 55–69 years) demonstrated poor agreement (Kappa coefficient: 0.13) **(Supplemental Table 2)**.

**Table 2:** Distribution and Kappa agreement of statin eligibility for primary and secondary prevention of cardiovascular disease according to the WHO-2019 and ACC/AHA-2018 guidelines.


**A. Primary prevention**		**Statin use eligibility based on ACC/AHA-2018 criteria, N (%)^a^**		**Kappa co-efficient**

		**Yes**	**No**	

**Statin use eligibility based on WHO-2019 criteria, N (%)^a^**	**Yes**	346 (34.7%)	0	0.46

	**No**	540 (65.3%)	1,811 (100%)	

**B. Secondary prevention**		**Statin use eligibility based on ACC/AHA-2018 criteria, N (%)^a^**		**Kappa co-efficient**

		**Yes**	**No**	

**Statin use eligibility based on WHO-2019 criteria, N (%)^a^**	**Yes**	443 (100%)	0	1.00

	**No**	0	0	


*Notes*: ^a:^ N = Number of participants; (%) = Column percentage (weighted). Kappa values were categorized as follows: <0.40 indicating poor to fair agreement, 0.41 to 0.60 as moderate agreement, 0.61 to 0.80 as substantial agreement, and 0.81 to 1.0 as almost perfect agreement.

**Figure 2:**
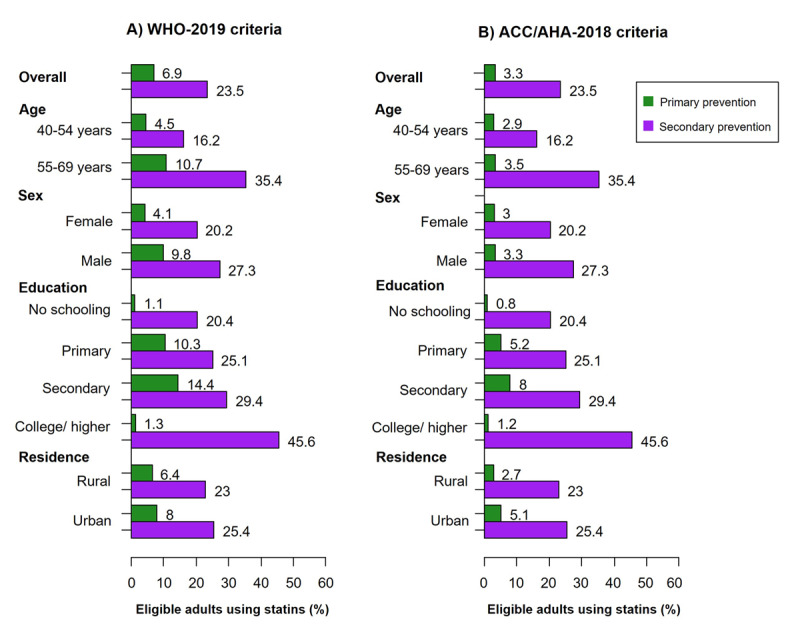
Proportion of individuals using statins among those eligible in each primary and secondary prevention group according to the WHO-2019 and ACC/AHA-2018 guidelines. Abbreviations: ACC, American College of Cardiology; AHA, American Heart Association; WHO, World Health Organization.

For primary prevention, among adults eligible according to the WHO-2019 guideline, only 6.9% (95% CI: 4.1–11.5) were using statins; while among those eligible according to the ACC/AHA-2018 guideline, only 3.3% (95% CI: 2.1–5.1) were using statins. For secondary prevention, 23.5% (95% CI: 16.9–31.6) of adults with a history of CVD were using statins. Older participants (aged 55–69 years) reported a higher proportion of statin use for both primary and secondary prevention compared to younger participants (aged 40–54 years). Additionally, male participants had a higher proportion of statin use than female participants. Statin use for secondary prevention was more common among participants with a higher education degree (college or higher) (45.6%, 95% CI: 20.3–73.5) compared to those with lower educational attainment (20.4%, 95% CI: 11.6–33.2) (Figure 2).

**Figure 3:**
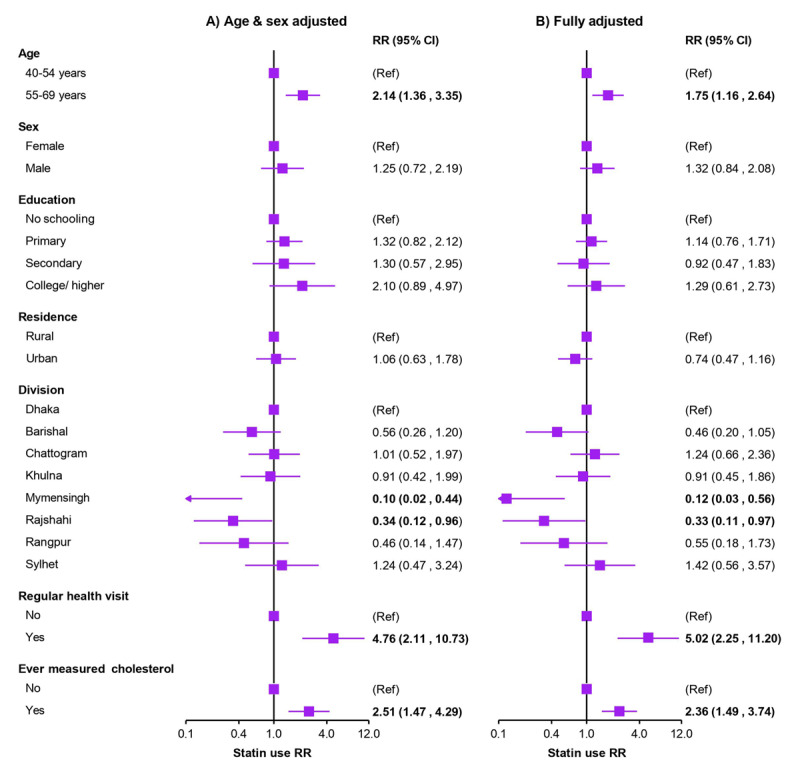
Modified Poisson regressions showing predictors of statin use among individuals eligible for statin therapy for secondary prevention of cardiovascular disease. • Results are presented as RRs (95% CIs), obtained using modified Poisson regressions and applying appropriate survey weights during analysis. Analyses were restricted to the secondary prevention outcomes only due to a very lower number of individuals using statins for primary prevention. Estimates with a P-value <0.05 are marked in bold. RR = risk ratio.

Both partially adjusted (adjusted for age and sex) and fully adjusted modified Poisson regression models were used to assess changes in effect sizes due to covariate adjustments. Although there were little variations in effect sizes between the two models, the direction of associations remained consistent. The modified Poisson regressions indicated that compared to younger age (aged 40–54 years), older age (aged 55–69 years) was associated with higher statin use for secondary prevention in both the partially adjusted model [Relative Risk (RR): 2.14; 95% CI: 1.36–3.35] and the fully adjusted model [RR: 1.75; 95% CI: 1.16–2.64] (Figure 3).

While not statistically significant, men were more likely than women to use statins [RR (fully adjusted): 1.32; 95% CI: 0.84–2.08]. Neither level of education nor place of residence (urban vs. rural) showed a significant association with statin use. However, compared to individuals residing in the Dhaka division, those living in the Mymensingh [RR (fully adjusted): 0.12; 95% CI: 0.03–0.56] or Rajshahi [RR (fully adjusted): 0.33; 95% CI: 0.11–0.97] divisions were at a higher risk of being non-users of statins. The strongest associations for statin use were observed among individuals who had regular health visits [RR (fully adjusted): 5.02; 95% CI: 2.25–11.20] and those who had their cholesterol levels measured at least once [RR (fully adjusted): 2.36; 95% CI: 1.49–3.74] (Figure 3).

## Discussion

This study provides the first comprehensive overview of the burden and predictors of statin use for both primary and secondary prevention of cardiovascular disease (CVD) in Bangladesh, using nationally representative data. We found that one in five adults and two in five adults in Bangladesh are eligible for statin therapy according to the WHO 2019 and the ACC/AHA 2018 guidelines, respectively. However, the prevalence of statin use among eligible individuals was markedly low, particularly for primary prevention, with only 6.9% and 3.3% of eligible individuals using statins according to the WHO 2019 and the ACC/AHA 2018 guidelines, respectively. Even among those eligible for secondary prevention, only about one in five individuals reported using statins. Statin use was generally higher among men, older individuals, those with higher educational attainment, those who had regular health visits, and those who had measured their cholesterol level at least once. Regional variations were also observed, with individuals residing in the Mymensingh and Rajshahi divisions being less likely to use statins.

Our results are consistent with existing evidence on the burden and use of statins in other settings. A recent cross-sectional study of 41 LMICs reported that, according to WHO-2019 criteria, approximately one in ten eligible individuals used statins for primary prevention, and about one in five eligible individuals used them for secondary prevention (9). Data from the Prospective Urban Rural Epidemiology study, conducted between 2003 and 2009, reported that only 4.3% of individuals from LMICs used statins for secondary prevention of CVD, compared to 66.5% in HICs (7). Our study adds to this body of evidence and substantially advances the understanding of statin use in an LMIC setting by providing a current estimate from Bangladesh based on the most recent international guidelines. To our knowledge, this is also the first study of its kind in South Asia, which could be adapted for use in other countries in the region to guide health system policies.

We also compared the total statin-eligible population using criteria from two different international guidelines and found that the ACC/AHA criteria would include approximately 18% more individuals due to its more conservative inclusion criteria compared to the WHO guidelines. A previous study conducted a head-to-head comparison of five major international guidelines for statin use from Europe, United States, and Canada, concluded that guidelines recommending statin use for a greater number of individuals would likely prevent more cardiovascular events than those recommending use by fewer individuals (21). Another study from the United States reported that 15.9% of adults would be newly eligible for statin therapy under the ACC/AHA guideline (22) compared to the Third Adult Treatment Panel guidelines, which had more flexible inclusion criteria (23). However, the broader eligibility criteria may also result in higher costs and resource use, which are significant considerations for LMICs like Bangladesh with constrained healthcare budgets. Therefore, a country-specific guideline for statin therapy is needed in Bangladesh, taking into account the efficacy, cost-effectiveness, healthcare infrastructure, and socio-economic context.

Consistent with previous studies, we found greater statin use among men, older individuals, and those with higher educational attainment (7,9,24). However, unlike earlier research, we did not observe any significant difference in statin use between individuals residing in urban and rural areas (7,9). The clinical benefits of statin therapy for cardiovascular risk reduction are similar for both men and women, and there are no sex-specific recommendations for statin eligibility or dosing (3,4). Therefore, future research should investigate the underlying reasons for the lower statin use among women in Bangladesh.

We also found that individuals who had regular health visits or had measured their cholesterol levels at least once were more likely to be on statin therapy. AHA has recommended that younger adults should measure their cholesterol and other lipid parameters every four to six years (25). However, only 6% of the Bangladeshi population has ever measured their cholesterol levels in a health facility, contributing to the low prevalence of statin use in the country. The cost of cholesterol measurement is typically higher than that of other cardiovascular risk assessments such as blood pressure, blood glucose, or body mass index, and this may discourage clinicians and individuals from conducting these tests (9). Policymakers in Bangladesh should address this issue by reducing the cost burden, potentially offering free cholesterol screening, and encouraging adults to visit health facilities regularly for cholesterol checks.

South Asia, including Bangladesh, currently bears one of the highest burdens of CVD globally (2), and therapeutic interventions like statins are particularly important in these settings to reduce the CVD burden. South Asians are more likely to develop cardiovascular risk factors such as diabetes, dyslipidemia, and hypertension at a younger age, and they are the only ethnic group that has been identified by the AHA as a “risk-enhancing factor” for CVD (6,26). Therefore, effective use of statins could potentially prevent thousands of CVD-related deaths and reduce associated healthcare costs in Bangladesh. It has been reported that lowering LDL cholesterol with effective statin therapy for five years could prevent major cardiovascular events in approximately 1,000 (10%) individuals out of 10,000 at higher risk of CVD (4).

Several other factors may also contribute to the underutilization of statins in LMICs, including Bangladesh. First, there are widespread misconceptions among clinicians and patients regarding the side effects of statins, which can lead to hesitancy in prescribing or taking these medications (27). The most common side effects that have been reliably demonstrated are myopathy (occurring in less than 1% of participants) and a modest risk of new-onset diabetes mellitus (approximately one case per thousand patients) (4,27). Consequently, expert panels have unequivocally recommended that the cardiovascular benefits of statin therapy far outweigh the risks of adverse effects (4,6,27,28). Second, most individuals, particularly those in the primary prevention category, are asymptomatic, and the effects of treatment are not immediately visible, making it easier to disregard statin therapy (29). Third, compared to other cardiovascular preventive drugs, such as antihypertensive and antidiabetic medications, statins are less affordable and not as widely recognized (30). Improving affordability and access, as well as educating clinicians and the public about the benefits of statin therapy, is crucial to increasing statin use in Bangladesh. In the future, qualitative research should be conducted to better understand the barriers to statin use in different LMIC settings, including Bangladesh. Additionally, it would be beneficial to examine the impact of targeted interventions designed to increase statin use in underserved populations.

A key strength of this study is its use of the most up-to-date nationally representative data, providing a comprehensive overview of statin use in Bangladesh. It also uses the most current international guidelines to identify the eligible population for statin therapy. However, some limitations should be noted. First, the cross-sectional nature of the study limits our ability to infer causality between the predictors and statin use for secondary prevention. Second, residual confounding may persist due to unmeasured confounders or inaccuracies in measured confounders. Third, the reliance on self-reported data may introduce recall bias or inaccuracies in reporting medication use. However, previous studies have reported a high degree of accuracy in self-reported cardiovascular disease history and medication use (7,31). Finally, we were unable to identify predictors of statin use for primary prevention because of the extremely small number of individuals using statins for primary prevention.

## Conclusions

In summary, our study highlights a substantial underuse of statins in Bangladesh, despite a considerable proportion of the population meeting the eligibility criteria according to current guidelines. Only one in twenty eligible individuals use statins for the primary prevention of CVD, while one in five do so for secondary prevention. Given the high prevalence of CVD in South Asian populations and the well-established association between South Asian ethnicity and an increased risk of CVD, it is imperative that healthcare professionals adopt a more flexible approach to statin inclusion criteria. Statin use should be encouraged for a broader group of at-risk individuals, particularly in low- and middle-income countries (LMICs), where the cost of cardiovascular interventions is often unaffordable, compared to more affordable therapeutic options. Policymakers should also focus on implementing strategies to increase statin use and ensure equitable access to these cost-effective interventions, which could substantially reduce the burden of CVD in Bangladesh and similar contexts.

## Data Accessibility Statement

This study used data from datasets of WHO STEPS 2018 Bangladesh survey, which are available from the WHO NCD microdata repository website. Approved researchers can request to this data through an application (https://extranet.who.int/ncdsmicrodata/index.php/home).

## Additional File

The additional file for this article can be found as follows:

10.5334/gh.1412.s1 Supplementary Materials. Supplemental Figure 1, Tables 1 to 2 and STROBE Checklist.

## Figures and Tables

**Table:** 

**Abbreviation**	**Full Description**
CVD	Cardiovascular Disease
ACC	American College of Cardiology
AHA	American Heart Association
WHO	World Health Organization
BP	Blood Pressure
LDL-C	Low-Density Lipoprotein Cholesterol
CI	Confidence Intervals
SD	Standard Deviation
LMIC	Low- and Middle-Income Countries
HIC	High-Income Countries
