# Supplementary material for: Burden and Predictors of Statin Use for Primary and Secondary Prevention of Cardiovascular Disease in Bangladesh: Evidence from a Nationally Representative Survey
Source: Glob Heart. 2025 Mar 12;20(1):28. doi: 10.5334/gh.1412 (PMC11908426; doi:10.5334/gh.1412)
Supplement: Supplementary Materials. — Supplemental Figure 1, Tables 1 to 2 and STROBE Checklist. [file gh-20-1-1412-s1.pdf]

Supplemental Material

Contents

Supplemental Figure 1. Flow chart illustrating selection of analytical dataset (n=3,140). 2

Supplemental Table 1. Statin eligibility criteria according to WHO-2019 and ACC/AHA-2018 guidelines for adults aged 40-69 years. 3

Supplemental Table 2. Distribution and kappa agreement of statin eligibility for primary prevention of cardiovascular disease according to WHO-2019 and ACC/AHA-2018 guidelines, by age group & sex 4

Supplemental Figure 1. Flow chart illustrating selection of analytical dataset (n=3,140).

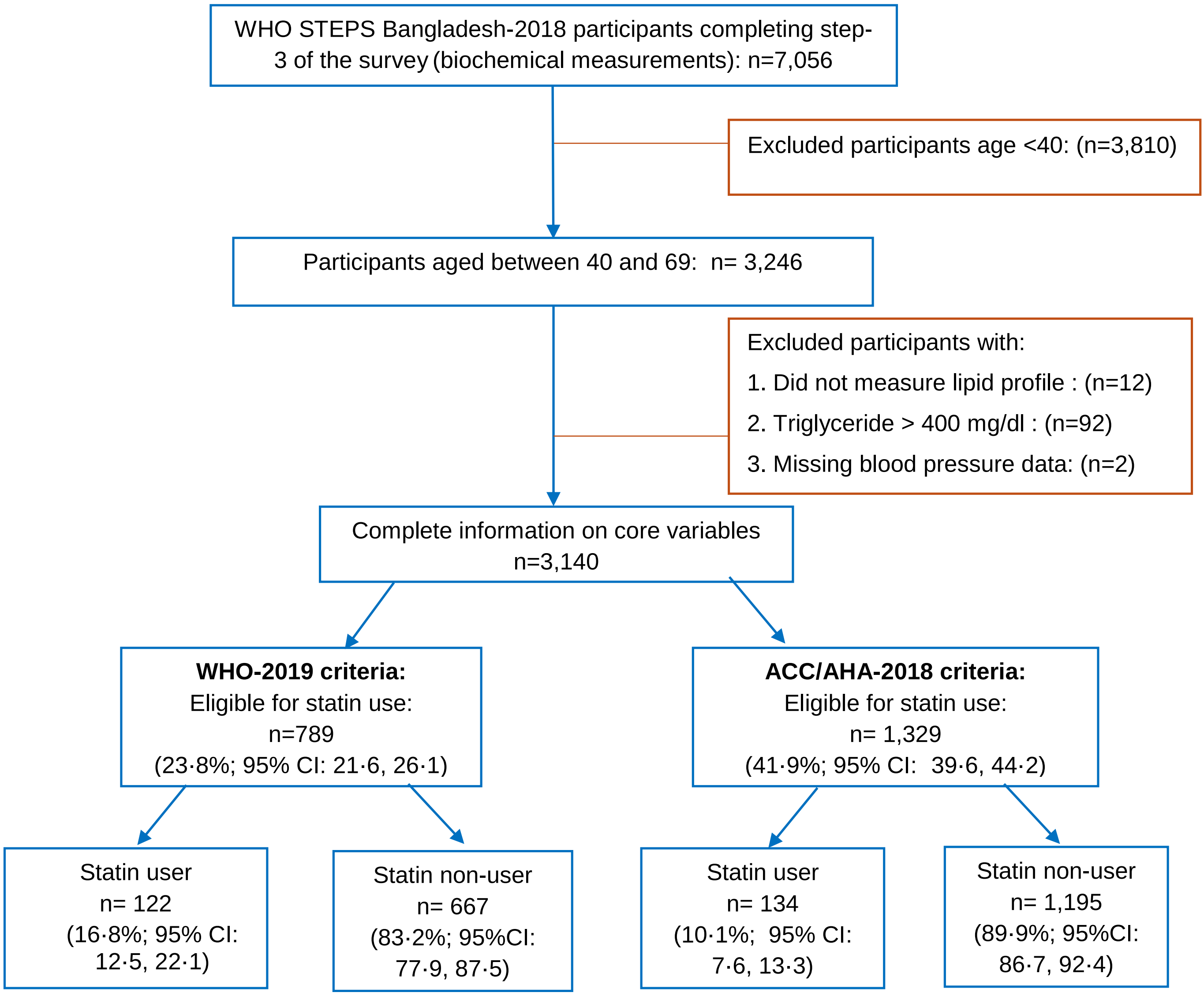

Abbreviations: ACC, American College of Cardiology; AHA, American Heart Association; WHO, World Health Organization.

Supplemental Table 1. Statin eligibility criteria according to WHO-2019 and ACC/AHA-2018 guidelines for adults aged 40-69 years.

**Prevention category**	**WHO-2019**	**ACC/AHA-2018**
**Primary prevention**	1. Type 2 diabetes mellitus<br>2. A 10-year cardiovascular disease risk of more than 20% using the 2019 WHO laboratory-based risk equations.[a]	1. LDL-C level ≥190 mg/dL<br>2. Type 2 diabetes mellitus<br>3. LDL-C level ≥70 - <190 mg/dL and predicted 10-year risk of any cardiovascular disease ≥7·5% using pooled cohort equation.[b,c]
**Secondary prevention**	Individuals with previous history of cardiovascular disease.	Individuals with previous history of cardiovascular disease.

[a]WHO-2019 laboratory-based risk model for cardiovascular disease is based on age, sex, smoking status, systolic blood pressure, history of diabetes, and total cholesterol.

[b]Pooled cohort equation for 10-year risk of cardiovascular disease is based on age, sex, smoking status, ethnicity, systolic blood pressure, history of blood pressure medication, history of diabetes, total cholesterol and HDL-C levels.

[c]South Asian ancestry are considered as a risk enhancer group in the ACC/AHA-2018 criteria. Thus, all Bangladeshi participants with LDL-C level ≥70 - <190 mg/dL and predicted 10-year risk of CVD ≥7·5% were considered eligible for statin therapy.

Abbreviations: ACC, American College of Cardiology; ASCVD, atherosclerotic cardiovascular disease; AHA, American Heart Association; PCE, pooled cohorts equations.

Supplemental Table 2. Distribution and kappa agreement of statin eligibility for primary prevention of cardiovascular disease according to WHO-2019 and ACC/AHA-2018 guidelines, by age group & sex

A. Female:

**Statin use eligibility based on WHO-2019 criteria, N (%)[a]**	**Statin use eligibility based on ACC/AHA-2018 criteria, N (%)[a]**		**Kappa co-efficient**
**1. 40-54 years**	Yes	No	0·90
Yes	115 (86·6%)	0	
No	21 (13·4%)	846 (100%)	
**2. 55-69 years**	Yes	No	0·55
Yes	42 (48·3%)	0	
No	50 (51·7%)	231 (100%)	

B. Male:

**Statin use eligibility based on WHO-2019 criteria, N (%)[a]**	**Statin use eligibility based on ACC/AHA-2018 criteria, N (%)[a]**		**Kappa co-efficient**
**1. 40-54 years**	Yes	No	0·45
Yes	101 (36·0%)	0	
No	170 (64·0%)	603 (100%)	
**2. 55-69 years**	Yes	No	0·13
Yes	88 (16·9%)	0	
No	299 (83·1%)	131 (100%)	

Notes: [a:] N = Number of participants; (%) = Column percentage (weighted). Kappa values were categorized as follows: <0·40 indicating poor to fair agreement, 0·41 to 0·60 as moderate agreement, 0·61 to 0·80 as substantial agreement, and 0·81 to 1·0 as almost perfect agreement.

STROBE Statement—Checklist of items that should be included in reports of ***cross-sectional studies***

	Item No	Recommendation	Page no
**Title and abstract**	1	(*a*) Indicate the study's design with a commonly used term in the title or the abstract	01-02
		(*b*) Provide in the abstract an informative and balanced summary of what was done and what was found	02
**Introduction**			
Background/rationale	2	Explain the scientific background and rationale for the investigation being reported	04
Objectives	3	State specific objectives, including any prespecified hypotheses	04-05
**Methods**			
Study design	4	Present key elements of study design early in the paper	05
Setting	5	Describe the setting, locations, and relevant dates, including periods of recruitment, exposure, follow-up, and data collection	05-06
Participants	6	(*a*) Give the eligibility criteria, and the sources and methods of selection of participants	05-06, supplemental figure 1
Variables	7	Clearly define all outcomes, exposures, predictors, potential confounders, and effect modifiers. Give diagnostic criteria, if applicable	06-07
Data sources/ measurement	8*	For each variable of interest, give sources of data and details of methods of assessment (measurement). Describe comparability of assessment methods if there is more than one group	05-07
Bias	9	Describe any efforts to address potential sources of bias	05-07
Study size	10	Explain how the study size was arrived at	05-07, supplemental figure 1
Quantitative variables	11	Explain how quantitative variables were handled in the analyses. If applicable, describe which groupings were chosen and why	06-08
Statistical methods	12	(*a*) Describe all statistical methods, including those used to control for confounding	06-08
		(*b*) Describe any methods used to examine subgroups and interactions	06-08
		(*c*) Explain how missing data were addressed	06-08
		(*d*) If applicable, describe analytical methods taking account of sampling strategy	-
		(*e*) Describe any sensitivity analyses	-
**Results**			
Participants	13*	(a) Report numbers of individuals at each stage of study—eg numbers potentially eligible, examined for eligibility, confirmed eligible, included in the study, completing follow-up, and analysed	07, 08, Supplemental Figure 1
		(b) Give reasons for non-participation at each stage	-
		(c) Consider use of a flow diagram	Supplemental Figure 1
Descriptive data	14*	(a) Give characteristics of study participants (eg demographic, clinical, social) and information on exposures and potential confounders	07-08

		(b) Indicate number of participants with missing data for each variable of interest	Supplemental Figure 1
Outcome data	15*	Report numbers of outcome events or summary measures	08-09
Main results	16	(*a*) Give unadjusted estimates and, if applicable, confounder-adjusted estimates and their precision (eg, 95% confidence interval). Make clear which confounders were adjusted for and why they were included	08-09
		(*b*) Report category boundaries when continuous variables were categorized	-
		(*c*) If relevant, consider translating estimates of relative risk into absolute risk for a meaningful time period	-
Other analyses	17	Report other analyses done—eg analyses of subgroups and interactions, and sensitivity analyses	08-09
**Discussion**			
Key results	18	Summarise key results with reference to study objectives	09-10
Limitations	19	Discuss limitations of the study, taking into account sources of potential bias or imprecision. Discuss both direction and magnitude of any potential bias	11
Interpretation	20	Give a cautious overall interpretation of results considering objectives, limitations, multiplicity of analyses, results from similar studies, and other relevant evidence	09-11
Generalisability	21	Discuss the generalisability (external validity) of the study results	11-12
**Other information**			
Funding	22	Give the source of funding and the role of the funders for the present study and, if applicable, for the original study on which the present article is based	12

*Give information separately for exposed and unexposed groups.
